# Barriers to Inter-Organisational Collaboration in the Preoperative Management of Patients with Osteoarthritis of the Hip or Knee

**DOI:** 10.5334/ijic.6995

**Published:** 2023-04-06

**Authors:** Mohsen Hussein, Nevenka Kregar Velikonja, Karmen Erjavec

**Affiliations:** 1 University of Novo mesto, SI; 2 Artros, Ljubljana, SI

**Keywords:** inter-organisational collaboration, barriers, integrated clinical pathway

## Abstract

**Introduction:**

Integrated clinical pathways should provide the best and most efficient treatment. As no study on barriers to inter-organisational collaboration has investigated the barriers to unimplemented integrated clinical care in a country with less efficiently organised health system, the study aimed to identify these barriers in the preoperative management of patients with hip or knee osteoarthritis in Slovenia.

**Methods:**

A cross-sectional study was conducted using multiple methods, including a quantitative survey with participants involved in target patient groups, and in-depth interviews with involved key actors at micro, meso and macro levels in Slovenia.

**Results:**

Respondents predominantly expressed a lack of inter-organisational collaboration. The exposed barriers are individualistic culture, the level of development of the health system, financing, administration, and regulatory frame at the macro level, shortage of staff at the meso level, and the lack of technological standards, trust, communication, and perception of pressures at the micro level.

**Discussion and conclusion:**

In addition to the barriers identified in previous studies, our study shows that individualistic culture and the level of development of the health system at the macro level, manifested as a pressure on health professionals and other actors at the micro level, are important barriers to inter-organisational collaboration.

## Introduction

To improve the integration of healthcare services, inter-organisational collaboration between healthcare settings, defined as the set of processes involving healthcare professionals representing multiple organisations when they work together in patient care [1], has recently become increasingly important, especially due to limited financial resources, ageing populations and comorbid chronic diseases [2,3,4,5,6]. Indeed, integration can help to coordinate previously separate healthcare delivery tasks across organisational boundaries [4,7], achieving benefits such as quality improvement, increased system efficiency and cost reduction, higher client satisfaction and better access to healthcare [2,8]. However, a literature review reveals several barriers that seem to hinder the emergence and development of such collaboration [5,6]. It is important to understand the barriers to the development of inter-organisational collaborative relationships, as this may help to explain and address the slow progress and limited efficiency and effectiveness of some inter-organisational collaborations in health care, and thus promote the successful implementation of integrated care in such settings [5,6].

Based on a patient-centred view of integrated care, Kodner and Spreeuwenberg’s [2] well-known definition of integration in healthcare, this is a coherent set of methods and models at the financing, administrative, organisational, service delivery and clinical levels that aim to create linkage, alignment and collaboration between healthcare sectors to improve quality of care and quality of life, consumer satisfaction and system efficiency for patients with complex, long-term problems involving multiple services, providers and settings. Auschra’s systematic literature review [6] identified twenty types of barriers, which were classified into six groups: i) barriers related to administration and regulation; ii) barriers related to funding; iii) barriers related to inter-organisational domain that include lack of leadership and coordination, differences in collaboration design and aims, incompatible organisational structures, lack of actors, and power imbalance and conflict; iv) barriers related to organisational domain that include cultural distance, previous experience in collaboration, experience and organisational vs. collective interests; v) barriers related to service delivery based on the lack of mutual understanding, lack of technical standards, lack of communication, differences in professionalisation and resistance to change; and vi) barriers related to clinical practice that include the lack of information exchange and confidentiality issues. In this literature review [6], the barrier ‘different professionalisation’ was mentioned most often, followed by ‘lack of leadership and coordination’ and ‘organisational vs. collective interests’. However, this interpretation could be biased, as the range of cases included, – inter-organisational collaboration in less developed countries – was largely omitted [6]. The review also showed that previous studies identifying individual barriers were mainly based on a qualitative methodological approach neglecting the patient’s perspective, and that none of these studies gave reasons for the failure of collaboration. In contrast to the prevailing studies conducted in developed countries, this study was performed in a post-socialist EU country with less efficiently organised health system.

Slovenia is a post-socialist country with 2.1 million inhabitants and a centralised health system with compulsory social insurance. Although health expenditure per capita has increased slightly in recent years, it is still far below the EU average. Public financing of the health system accounted for 73% of health expenditure in 2019 [9]. Healthcare is mainly provided by public health centres. The capitation system is implemented at the primary level, general practitioners (GPs) play an important role as gatekeepers [10,11]. The analysis of health system in Slovenia revealed relatively lower efficiency of the health system compared to OECD countries [12]. The Slovenian health and social care system, similar to other Central and Eastern European health care systems (e.g. Croatian, Czech, Slovak and Polish) underwent significant changes during the transition period. The recognition of the barriers to inter-organizational collaboration in Slovenia, such as the lack of culture of cooperation, a large deficit in public funding of health care systems, and a lack of sufficient human resources (GPs, nurses, other health professionals) [13] suggests such barriers in other Central and Eastern European countries as well.

This study focused on the preoperative management of patients with hip or knee osteoarthritis. Surgical treatment and rehabilitation of hip and knee osteoarthritis are organised in relatively well-defined clinical pathways. Evidence-based clinical pathways have been shown to improve health-related quality of life (HRQoL) in hip and knee arthroplasty patients with degenerative joint disorder in routine clinical practice [14]. However, it has been noted that the efficient use of preoperative diagnostics and conservative treatment needs further improvement [15,16]. Preoperative management of patients with hip and knee osteoarthritis should focus on preparation for arthroplasty in terms of improving the prognosis of surgical treatment, focusing on different aspects of the patient’s psychophysical state [17]. Studies show that the success of surgical treatment is better in patients who are well prepared for surgery. In this context, rehabilitation programmes that increase muscle strength and mobility [18], and nutrition counselling [19] have significant influence on pain relief and postoperative functionality of patients with osteoarthritis, while preoperative education is mainly associated with lower preoperative anxiety [20]. In a broader context, preoperative involvement of other specialists (e.g. cardiologists, pulmonologists) is important to prevent severe complications, especially in the elderly. Integrated clinical pathways involving different professional groups, e.g. physiotherapists, occupational therapists, dieticians and psychologists, who are also involved in the conservative management of patients with hip and knee osteoarthritis, as proposed by van den Bogaart [16], are successfully implemented in some countries, but poorly implemented in Slovenia.

Since shared decision-making is a key component of patient-centred care that takes into account clinical records and patient preferences and values [21], and since no study on the barriers to inter-organisational collaboration involved patients and used a multiple-methods approach, has examined the barriers to implementation of integrated clinical pathways in a country with less efficiently organised health system [12], this study aimed to identify the barriers to integrated care in inter-organisational collaboration in the preoperative management of patients with hip or knee osteoarthritis in Slovenia.

## Methods

To obtain a comprehensive overview of Slovenian inter-organisational collaboration in the preoperative management of patients with hip or knee osteoarthritis, we conducted a cross-sectional study using multiple methods. We first used a quantitative survey, which gave us a broad overview based on statistical analysis, and through qualitative research we obtained more detailed and emotionally driven insights on detected issues. Due to the Covid-19 pandemic, the study was performed in two phases between June and October 2021 and April and June 2022.

### Quantitative approach

Data were collected by an online survey to determine the performance of collaboration between different healthcare providers in the preoperative management of patients with hip or knee osteoarthritis. All medical and other professionals involved in the preoperative management of patients with hip or knee osteoarthritis [17,18,19,20] were invited to participate in the study, namely physicians, nurses, other health professionals (physiotherapists, dieticians occupational therapists and psychologists), and administrative staff in various Slovenian healthcare facilities, including public general hospitals (orthopaedic departments), private orthopaedic clinics, specialised public orthopaedic or rehabilitation centres and community health centres. Then a list of potential participants was created. Their e-mail addresses were obtained from public websites. The participants were invited by an email with a link to the online survey, which included a brief description of the research purpose and objectives. The participants were informed that they agreed to participate in the study by completing the questionnaire. Three reminders were sent one week apart to increase participation; 94 questionnaires were fully completed (Table 2). Completing the survey took about 10 minutes. To avoid any risk that might be associated with participation in this survey, the researchers assured that participants’ responses could not be identified. No participant identifiers were collected, the responses were only used to compile statistics. To develop the instrument, we adapted the measures for all study variables from previously published studies [21,22,23]. To further refine the measurement items from the study construct, we conducted interviews with 5 academics and 5 healthcare workers with the experience in inter-organisational collaboration. We also conducted a pilot study with a sample of 25 health workers. The first question measured the frequency of inter-organisational collaboration and included three statements on a five-point scale from 1 – ‘Never’ to 5 – ‘Very often’ (Table 3). The second set explored the assessment of the performance of inter-organisational collaboration. It included seven statements that participants rated on a five-point scale from 1 – ‘I do not agree at all’ to 5 – ‘I totally agree’ (Table 4). The third set, which included socio-demographic variables, contained five questions on gender, age, educational level, and profession. The questionnaire exhibited a very high degree of internal consistency (Crombach’s alpha was 0.845).

Descriptive analysis, and the Kruskal-Wallis one-way analysis of variance (ANOVA) were used for testing whether there was a statistically significant difference between the assessment of inter-organisational collaboration among four groups of respondents (physicians, nurses, other health professionals and others). A P value < 0.05 was considered statistically significant. The data were analysed with SPSS, version 25.0 (IBM Corp, Armonk, NY, USA).

### Qualitative study

In order to obtain a comprehensive overview of the barriers to inter-organisational collaboration in the preoperative management of patients with hip or knee osteoarthritis in Slovenia, a multi-level qualitative approach was used: patients and health professionals were interviewed to obtain insight at the micro-level, the community and other health organisations at the meso-level, and the national level professionals at the macro level. At the meso-level, participants were selected based on their leading role in the organisations, and at the macro level based on their role in the health system (regulatory, financial, professional, and scientific stakeholders). At the micro-level, the inclusion criterion for patients with hip or knee osteoarthritis was the ability to communicate verbally; for health professionals, the inclusion criterion was employment in the primary healthcare sector, secondary public and private health organisations or rehabilitation centres (Table 1).

**Table 1:** Qualitative data collection characteristics (N = 53).


LEVEL OF HEALTHCARE SYSTEM	PARTICIPANTS

**Micro**	16 patients

	10 physicians-specialists (6 orthopaedists, 2 anaesthetists and 2 physiatrists)

	5 general practitioners

	4 nurses

	4 physiotherapists

	3 other health professionals (dietician, occupational therapist and psychologist)

**Meso**	6 managers of health organisations

**Macro**	5 stakeholders from regulatory, financial, professional and scientific sectors


A thematic interview guide was developed for data collection, based on the barriers to inter-organisational collaboration found in literature reviews and on contextual knowledge. The main themes were the assessment of the performance of inter-organisational collaboration and the barriers to the implementation of the integrated clinical pathway of preoperative management of patients with hip or knee osteoarthritis. As the data collection progressed, further relevant key information was identified by snowballing and added to the list of participants until saturation was reached (no new information was added). All interviews were conducted in person or online due to Covid-19 pandemic measures by six experienced researchers. The in-depth interviews, which lasted on average about 60 minutes, were recorded with the prior consent of the participants. Anonymised statements were transcribed. The data were analysed using thematic analysis. In order to determine when data saturation occurred, the thematic analysis was conducted in an iterative cycle simultaneously with data collection. In the first step, the transcribed texts were usually read several times and descriptive notes were made on the content. Then a second reading was done to code the data, i.e. to mark phrases or sentences and add shorthand or codes describing their content. When patterns were identified among the codes, similar codes were combined to generate a theme. We reviewed the themes by returning to the transcribed texts and checking whether the themes represented the content. The themes were concise and easy-to-understand names based on the barriers identified by Auschra in her systematic literature review of barriers to the integration of care in inter-organisational settings [6]. The analysis of each interview was conducted by two independent researchers. Any problems with data analysis and coding were discussed by the steering committee and resolved by consensus.

### Ethical consideration

Data collection was part of the project ‘Impact of integrated clinical pathways on patient outcomes, communication and cost-effectiveness’ funded by the Slovenian Research Agency (No. L7-2631-3824-2020). The research was approved by the National Committee of Medical Ethics of the RS (No. 0120-189/2021/3).

## Results

### Results of quantitative study

The questionnaire was completed by nurses (50.0%), physicians (31.9%), other healthcare professionals (physiotherapists, clinical pharmacists, clinical dietitians, psychologists, social workers, hygienist) (11.7%) and others such as health administrators (6.4%). The sample was dominated by women (73.4%) and respondents with BSc education (39.4%). Most respondents were employed in public sector, as healthcare in Slovenia is predominantly carried out in public sector [10]; specifically, they were employed in general hospitals as osteoarthiris is predominantly treated in these institutions (Table 2). The mean respondent age was 40 years.

**Table 2:** Characteristics of participants (N = 94).


CHARACTERISTICS	N	%

**Gender**		

Male	25	26.6

Female	69	73.4

**Level of education**		

Secondary school	17	18.1

Bachelor’s degree	37	39.4

Specialisation and master’s degree	29	30.9

Doctorate	11	11.7

**Professional groups**		

Nurses	47	50.0

Physicians	30	31.9

Other healthcare professionals	11	11.7

Others	6	6.4

**Institution of employment**		

Community health centre	10	10.6

Public general hospitals	70	74.5

Public orthopaedic or rehabilitation centre	5	5.3

Private orthopaedic centre	9	9.6


ANOVA analysis of different professional groups (Tables 3 and 4) shows a low level of collaboration among organisations, and a varied evaluation of different statements according to the professional group. Table 3 shows that the statement about inter-organisational collaboration was rated relatively low, meaning that the professionals from other organisations involved in the medical treatment of a patient were rarely team members, although they more often relied on the received documentation and also consulted other competent persons in making decisions. Bonferroni post-hoc test showed that in all the statements where ANOVA revealed differences among groups at the 0.01 level of significance, there was a significant difference in the evaluation of statements between medical doctors and nurses; in all cases nurses rated the statements higher than doctors and other professional groups. All three statements were rated lowest by other (non-HP) employees, as they were least involved in team coordination of patient treatment.

**Table 3:** Assessment of the frequency of collaboration with other professions in the preoperative management of a patient with hip or knee osteoarthritis. (1 ‘never’, 2 ‘rarely’-in less than 20% of patients’, 3 ‘occasionally’- in 20% to 50% of patients, 4 ‘often’- in 50% to 80% of patients, 5 ‘very often’ – in more than 80% of patients).


STATEMENT	PROFESSION	N	MEAN	SD	ANOVA (P)

**To monitor the patient’s medical condition, I rely on the received documentation (on the authenticity of the documentation as a basis for clinical treatment).**	MD	30	3.27	1.82	0.002**

	nurse ↑	47	4.21	1.28	

	other HP	11	3.55	2.50	

	other employees ↓	6	1.67	1.63	

	all respondents	94	3.67	1.77	

**When making decisions, I ask for an opinion another competent person.**	MD	30	2.60	1.73	<0.001**

	nurse ↑	47	4.06	1.23	

	other HP	11	2.36	2.11	

	other employees ↓	6	2.33	2.06	

	all respondents	94	3.29	1.70	

**All those involved in the medical treatment of an individual patient (including professionals from other organisations) are treated as team members.**	MD	30	2.20	1.64	0.031**

	nurse ↑	47	2.64	1.53	

	other HP	11	1.36	1.80	

	other employees ↓	6	1.17	0.41	

	all respondents	94	2.26	1.61	


Legend: ** Differences among groups significant at the 0.01 level; ↓ – lowest mean score; ↑ – highest mean score; MD – physicians; HP – health professionals.

**Table 4:** Respondents’ opinions about the performance of collaboration between their organisation and other healthcare providers in treatment of patients with hip or knee osteoarthritis (from 1 ‘I absolutely don’t agree’ to 5 ‘I absolutely agree’).


STATEMENTS	PROFESSION	N	MEAN	SD	ANOVA (P)

We are sufficiently informed about the competencies of other healthcare organisations and support activities according to the needs of the patients we treat.	MD ↑	30	3.60	0.77	0.001**

	nurse	47	2.43	1.31	

	other HP	11	2.18	2.35	

	other employees ↓	6	1.50	1.22	

	all respondents	94	2.71	1.46	

We provide continuous patient care in collaboration with external healthcare providers.	MD	30	2.33	1.12	0.009**

	Nurse ↑	47	2.74	1.56	

	other HP ↓	11	1.27	1.48	

	other employees	6	1.50	1.22	

	all respondents	94	2.36	1.48	

External healthcare providers are responsive in engaging in patient care.	MD	30	2.23	1.10	0.009**

	Nurse ↑	47	2.72	1.44	

	other HP ↓	11	1.27	1.48	

	other employees	6	1.67	1.63	

	all respondents	94	2.33	1.42	

Depending on the patient’s needs, we exchange information with external providers for more comprehensive, safe, and quality treatment.	MD	30	2.23	1.13	0.053

	Nurse ↑	47	2.60	1.52	

	other HP ↓	11	1.45	1.63	

	other employees	6	1.50	1.22	

	all respondents	94	2.28	1.03	

We are more cost-effective through targeted collaboration with external healthcare providers.	MD	30	2.03	1.44	0.016*

	Nurse ↑	47	2.60	1.51	

	other HP ↓	11	1.18	1.66	

	other employees	6	1.67	1.63	

	all respondents	94	2.19	1.46	

We have appropriate legal bases for collaboration with external healthcare providers.	MD	30	1.97	0.99	0.013*

	nurse ↑	47	2.43	1.52	

	other HP↓	11	1.00	1.41	

	other employees	6	1.50	1.22	

	all respondents	94	2.05	1.41	

We have secured funding for collaboration with external healthcare providers.	MD ↑	30	1.77	0.85	0.259

	nurse	47	1.64	2.07	

	other HP↓	11	1.00	1.41	

	other employees	6	1.67	1.63	

	all respondents	94	1.61	1.10	


Legend: * Differences among groups significant at the 0.05 level; ** differences among groups significant at the 0.01 level; ↓ lowest mean score; ↑ highest mean score; MD – physicians; HP – health professionals.

Table 4 shows that the respondents mostly disagreed with various aspects of collaboration with other healthcare providers. The highest rated statement was, ‘We are sufficiently informed about the competencies of other healthcare organisations/providers and support activities according to the needs of the patients we treat’, as physicians predominantly agreed with this statement. However, no other statement was rated more than 3.0 on average in any of the professional group. The lowest rated statement was the statement about funding sources for collaboration with other healthcare providers. The Bonferroni post-hoc test showed that there was a significant difference between physicians and nurses in the evaluation of being informed about the competencies of other healthcare organisations and support activities, where physicians perceived themselves as better informed than nurses and others. The difference in the rating of other statements between physicians and nurses was not significant.

### Results of qualitative study

As the results of quantitative study revealed a lack of inter-organisational collaboration, we tried to identify the barriers to inter-organisational collaboration in implementing an integrated clinical pathway of preoperative management of patients with hip or knee osteoarthritis (Table 5).

**Table 5:** Barriers of inter-organisational collaboration in treating a patient with hip or knee osteoarthritis.


BARRIERS – THEMES	PATIENTS	HEALTH PROFESSIONALS	OTHER STAKEHOLDERS

**Macro level**			

Individuality culture and level of developed System	Personal responsibility for/engagement in obtaining health treatment; Distrust in system	Poorly functioning health system with exceptional individuals	Low personal responsibility for introduction of key changes

Administrative/Regulative	Too much administration	Too much administration; Complicated and time-consuming implementation of regulations	Weak work organisation in health settings

Funding	Lack of resources	Lack of resources; unpaid inter-organisational collaboration	Lack of resources

Power imbalance and conflicts	–	Overpowering financier	–

**Meso level: (inter-)organisational level**			

Lack of leadership and coordination	–	Weak organisation; Inter-organisation collaboration on personal level	Organisational managers protect their territory/rights

Lack of staff	Shortage of GPs	Shortage of nurses and GPs	Shortage of nurses and GPs

**Micro level (service delivery)**			

Different professionalisation	–	Interdisciplinary rivalry	–

Lack of technological standards	–	Incompatible IT infrastructure	Incompatible IT infrastructure

Lack of trust	Lack of trust	Lack of trust	Lack of trust

Lack to communication	Lack of time for communication	Lack of time for communication	Lack of willingness to communication

Resistance to change	Fear of losing one’s rights	Fear of additional workload	Fear of losing status quo

**Clinical practices**			

Lack of information exchange	–	Lack of information exchange between GPs and specialists	–

Pressure	Demands on special health treatment	Pressure from patients	Application of pressure in general


#### Macro-level barriers

All participants pointed out the barrier based on the prevailing *culture of individuality*, which manifests itself in the thinking of the main actors to be primarily responsible for the realisation of their own needs and actions.

At the national level, individuality culture manifests itself in not taking personal responsibility for systemic measures by key authorities (e.g., governmental, insurance and health institutions) because they fear of being personally discredited by a failure of the system, ‘The system works badly because those in charge do not approve the proposed measures. They are afraid to take personal responsibility, as they will be blamed if the changes are not implemented well. And it is very likely that it will not work, and the blame would be addressed to the responsible person.’ (Stakeholder 1).

The perception of patients and health professionals is that the system is overworked and poorly functioning and does not support them so that they themselves have to take responsibility for organising the individual treatment process). A typical statement from a patient was, ‘Because I have to take care of everything myself, I had to make sure that I was operated on by a good orthopaedist in a relatively short time and not in two years.’ (Patient 1). Most health professionals expressed a similar focus on the individual and described the health sector (especially orthopaedics) as dominated by outstanding individuals,

‘Since the system is overloaded and offers little autonomy and development opportunities, it works on the principle of individual initiative. Those who have a sufficiently strong ego to overcome the organisational obstacles succeed. However, if they do not see their own benefits, innovations in preoperative management of patients in terms of inter-organisational collaboration will not be implemented.’ (Specialist 1).

All patients and health professionals agreed that a key barrier to the introduction of an integrated clinical pathway was *too high administrative burden*. All health professionals also pointed out the *complicated and time-consuming implementation of regulations*, ‘Additionally, the implementation of any regulations is too complicated, the bureaucracy takes too much time.’ (Specialist 2). Funder’s representatives disagreed, claiming that ‘they have greatly simplified and digitalised administrative procedures, which is not implemented in practice due to poor organisation of work in health organisations’. (Stakeholder 2).

All respondents also agreed that the biggest barrier was the *lack of resources* for healthcare. Health professionals stressed that inter-organisational collaboration was not systematically paid for, ‘We are not innovative in the first place because there is a lack of money and such collaboration is not paid for.’ (GP 1).

Another important barrier is the power imbalance and conflicts between health organisations and financiers: ‘The barrier is the superiority of the financier who has no idea about the *specifics of the profession*. The Slovenian Health Insurance Institute exercises control and does not listen to us, so we feel humiliated.’ (GP 2).

#### Meso-level barriers

More than half of the health professionals also stressed the *lack of leadership and coordination* by managers of health organisations, manifested as poor organisation of work and lack of involvement of health professionals in the management: ‘Yes, the problem is poor organisation of work and our exclusion from the decision-making process.’ (Specialist 3). This was the only point on which the managers of health organisations differed from other health professionals. They argued that they were managing the organisation as best as they could, but the system was obsolete, rigid and required an increase in funding: ‘We are performing miracles in a system that is outdated and inflexible and does not consider the real financial needs of healthcare.’ (Manager of a health organisation 1). National stakeholders on the other hand argued that health organisation managers are not only poorly organised but also protect their territory/rights: ‘There is a lot of room for improvement in the organisation of institutions, but they should not think about the benefits of their organisations.’ (Stakeholder 3). All participants agreed that the biggest barrier was the *lack of staff*:

‘This is the biggest problem of the whole health system, the lack of physicians and especially nurses. We are not paid enough and work too much; that is why most of my colleagues want to go elsewhere to work.’ (Nurse 1).

#### Micro-level barriers

*The difference in professionalisation* is an obstacle that manifests itself as professional rivalry among different professions. For example, ‘GPs should know and do basic diagnostics, and now they just refer patients to us’. (Specialist 4). On the other hand, GPs claimed that they were ‘overworked and pre-operative diagnostic should be done by specialists’ (GP 1). *Lack of technological standards* is another major barrier to innovation, as different organisations use different IT: ‘It is a disaster that we have different IT systems that are not compatible.’ (Specialist 5). *Lack of trust* between organisations manifests itself as a lack of trust in the health system and between organisations. Health professionals added that due to their distrust in the work of organisations and the system in general, they collaborate with other organisations at a personal level. For example, ‘They only trust me because they know me, and they refer the patients only to me for physiotherapy treatment’. (Other health professional 1). *Lack of communication* is another barrier mentioned by all respondents, due to lack of time, incompetence or personal characteristics: ‘They do not communicate enough because they do not have time, especially the nurses, and some doctors are too selfish to be friendly.’ (Patient 2).

*Resistance to change* is a barrier listed by most participants, as they fear that things will get worse: ‘I am afraid that we will lose what we have.’ (Patient 3). *Lack of information sharing* was also a frequently expressed barrier by health professionals due to lack of compatible IT and fear of unfair criticism from others, ‘I am very reluctant to pass on the information because of IT incompatibility. I also do not want to be exposed to undue criticism.’ (Specialist 6).

*Pressure from others* was highlighted by most participants and manifested in patients’ demands to be treated by a particular specialist only, perceived as extreme pressure by health professionals, and general pressure by representatives at the national level: ‘Everyone presses everyone, that is, patients, doctors, pharmacists, politicians and like everyone who wants something’ (Stakeholder 4).

## Discussion

The combination of qualitative and quantitative methods in our study was useful as it provided a broader and deeper insight into inter-organisational collaboration. The survey results show that collaboration among professionals from different health organisations is very rare. Comparison with the results of other studies that have implemented integrated clinical pathways in preoperative management of patients with hip or knee osteoarthritis shows that inter-organisational collaboration is well organised and effective in many countries [14,15,16,17,18,19,20,21,22,23,24,25], it can improve health related quality of life [14], however, the study in the Netherlands shows that implementation of the pathway had a positive effect on GPs’ diagnostic behaviour only in relation to the knee but not the hip [16]. The statements about inter-organisational collaboration and communication were rated higher by nurses than by physicians and others, they much more often relied on documentation and consulted other competent people, probably due to the fact that nurses are generally most involved in coordination of patient’s treatment.

Why is inter-organisational collaboration in Slovenia, a post-socialist European country, so weak in this health segment? The answer lies not only in the health segment or the weak health system, but also in the broader social context that influences the functioning of all major actors in the health system. The individualistic culture is an important barrier to the introduction of innovations. Interviewers described that patients in Slovenia come for an anaesthesiology examination one month before surgery, where other specialists are involved if necessary; this collaboration works well because it is usually within the same facility where the patient will be operated. Preoperative physiotherapy and dietetic consultations are rarely used due to communication barriers between professions and between institutions. There are individual good practices of preoperative education programmes where patients learn exercises for postoperative rehabilitation, and for walking with crutches, but these are rare initiatives. In such a system integrative pathways are not systematically implemented, outstanding individual health professionals develop and maintain personal collaboration with professionals from other organisations that provide health treatment at the same quality level. In this context, patients who do not trust in (the health) system think that they have to take care of themselves to receive appropriate medical treatment, which manifests itself at the micro level as a pressure on health professionals. However, at the national level, those in charge do not make important decisions because they fear of being personally attacked and blamed for a dysfunctional system which is understaffed, underfunded, and bureaucratised. The interviews reveal that individualism plays an important role in hindering the introduction of innovations. This is in line with the findings of a sociological/anthropological analysis that in a poorly functioning transitional social system with a strong individualistic culture, mistrust in the system and the main social authorities, personal interests prevail, which further hinders the development of different innovations all other levels [26]. The results of the in-depth interviews show that the barriers are not only related to the specifics of hip and knee osteoarthritis, but to the health system in general. This is not an isolated case, as a study in nine Central and Eastern European countries shows that an individualistic culture cannot or does not understand the culture of cooperation, which can be found in other post-socialist countries as well [13].

Another important barrier to the adoption of innovation is the power imbalance and conflicts between health organizations and financiers. GPs in particular complained of excessive control and sanction by the Health Insurance Institute. This is consistent with the studies in which about half of family medicine specialists in Slovenia [27] and other countries of the former Yugoslavia [28] reported excessive control and sanction by financing insurance agency.

The analysis also revealed seemingly contradictory data resulting from different views of the participant on the barriers to introducing innovations in the Slovenian health system. At the macro level, all health professionals interviewed mentioned too much paperwork imposed on them by the Health Insurance Institute and taking too much of their time as an important barrier, whereas the financiers’ representative said that the implemented digitalisation should have greatly simplify their work, however poor organisation of daily work of health professionals overrules these benefits. The study confirms that both perspectives are relatively true, namely that health professionals, especially GPs, have much paperwork to do [29], and that key Slovenian authorities at the macro and meso levels have not ensured digitalisation to be successfully implemented [30].

A comparison with the results of a literature review on the barriers to the integration of care in inter-organisational settings [6] shows that most of the barriers identified in other studies are consistent with this study, but certain barriers are less explicitly mentioned by participants because macro barriers prevailed. For example, the barriers of incompatible organisational structure, differences in the design and goals of collaboration, lack of mutual understanding and organisational vs. collective interests were implicitly expressed as they are less important due to a poorly functioning health system. The fact that macro barriers are important in a less developed health system is also evidenced by the finding that the power imbalance which other studies have identified at the meso level [6] is manifested at the macro level in Slovenia. Furthermore, no barrier was found in relation to the confidentiality of patient data, which is due to stricter laws, and confidentiality is respected. Our findings suggest that many reasons for the slow progress or even failure of integrated care across organisational boundaries can be found at the macro level.

The results suggest that no major changes can be expected in countries with less efficiently organised health system such as Slovenia unless systemic changes are initiated at the macro level, such as paying more attention to solving health problems, better financing of the health sector, employing more health professionals, appointing key people in positions of responsibility at the national and organisational levels, etc. Currently, most of these factors significantly deviate from other OECD countries and have been exposed as potential causes of less efficient health system organisation [12]. The importance of system organisation was also emphasised in the review by Leithaus et al. [31], which highlighted the role of coordinators in the success of the integrated approach, especially in the treatment of frail patients, which is also common in patients with hip or knee osteoarthritis. Although we tried to ensure the highest level of validity and credibility of the study, e.g. through triangulation, the use of multiple methods and the inclusion of different groups of stakeholders (health professionals, managers, financiers, regulators and professional and scientific participants on the one hand, and patients on the other), the involvement of different researchers in the conduct of the study and the analysis of the data by following the qualitative instructions for the conduct of the study, there are some limitations of the study, the most important of which is that the study was conducted in the unusual situation related to the Covid-19 pandemic, which affected organisation of work and collaboration. Although we tried to include as many GPs as possible in the quantitative survey, the response rate is despite repeated calls relatively low due to their overload. In addition, we would like to stress that although interviewees indicated that the barriers to inter-organisational collaboration apply to the entire healthcare system, the results only apply to the analysed case of preoperative management of patients with hip or knee osteoarthritis.

## Conclusion

As there is a research gap on the barriers to inter-organisational collaboration in a less developed EU country, which would investigate the not yet implemented integrated clinical pathway with quantitative and qualitative methods involving all key actors, this study shows that inter-organisational collaboration is very rare in case of preoperative management of hip or knee osteoarthritis in Slovenia and other Central and Eastern European countries. However, a more complex picture of barriers to the integration of care in inter-organisational settings emerges, as macro-level barriers are very important, such as individualistic culture and level of development of health systems, financing and administration, and regulatory barriers.
